# Overview of Lung Ultrasound in Pediatric Cardiology

**DOI:** 10.3390/diagnostics12030763

**Published:** 2022-03-21

**Authors:** Massimiliano Cantinotti, Pietro Marchese, Raffaele Giordano, Eliana Franchi, Nadia Assanta, Vivek Jani, Shelby Kutty, Luna Gargani

**Affiliations:** 1 Fondazione G. Monasterio CNR-Regione Toscana, 54100 Massa, Italy; pitrino91@gmail.com (P.M.); eliana.franchi@ftgm.it (E.F.); assanta@ftgm.it (N.A.); 2 Institute of Clinical Physiology, National Research Council, 56124 Pisa, Italy; 3 Adult and Pediatric Cardiac Surgery, Department of Advanced Biomedical Sciences, University of Naples “Federico II”, 80131 Napoli, Italy; r.giordano81@libero.it; 4 Taussig Heart Center, Department of Pediatrics, Johns Hopkins Hospital, Baltimore, MD 21205, USA; vpjani@ucsd.edu (V.J.); shelby.kutty@gmail.com (S.K.); 5 Department of Surgical, Medical and Molecular Pathology and Critical Care Medicine, University of Pisa, 56124 Pisa, Italy; lunagargani@gmail.com

**Keywords:** congenital, pediatric, echo, ultrasound, cardiac

## Abstract

Lung ultrasound (LUS) is increasing in its popularity for the diagnosis of pulmonary complications in acute pediatric care settings. Despite the high incidence of pulmonary complications for patients with pediatric cardiovascular and congenital heart disease, especially in children undergoing cardiac surgery, the use of LUS remains quite limited in these patients. The aim of this review is to provide a comprehensive overview and list of current potential applications for LUS in children with congenital heart disease, post-surgery. We herein describe protocols for LUS examinations in children, discuss diagnostic criteria, and introduce methods for the diagnosis and classification of pulmonary disease commonly encountered in pediatric cardiology (e.g., pleural effusion, atelectasis, interstitial edema, pneumothorax, pneumonia, and diaphragmatic motion analysis). Furthermore, applications of chest ultrasounds for the evaluation of the retrosternal area, and in particular, systematic search criteria for retrosternal clots, are illustrated. We also discussed the potential applications of LUS, including the guidance of interventional procedures, namely lung recruitment and drainage insertion. Lastly, we analyzed current gaps in knowledge, including the difficulty of the quantification of pleural effusion and atelectasis, and the need to differentiate different etiologies of B-lines. We concluded with future applications of LUS, including strain analysis and advanced analysis of diaphragmatic mechanics. In summary, US is an easy, accurate, fast, cheap, and radiation-free tool for the diagnosis and follow-up of major pulmonary complications in pediatric cardiac surgery, and we strongly encourage its use in routine practice.

## 1. Background

Lung ultrasound (LUS) is an ideal tool for the diagnosis and follow-up of pulmonary complications after pediatric cardiac surgery. LUS offers the possibility to monitor lung disease progression easily and quickly at the patient’s bedside, and it allows us to evaluate the response to medical therapy (i.e., diuretics) and physiotherapy [1,2,3,4]. In addition, several potential applications of LUS exist, particularly for children after cardiac surgery. In this setting, LUS can be employed for the diagnosis of post-op lung complications, including atelectasis, effusion, lung congestion, pneumonia, pneumothorax, obstructive pulmonary disease, and diaphragmatic motion anomalies [1,2,3,4]. Compared to traditional chest radiography, LUS allows for the differential diagnosis of many common pulmonary complications after pediatric cardiac surgery [1,2,3,4]. For instance, LUS easily differentiates between effusion and atelectasis, both of which are common sequelae of cardiac surgery, and importantly, require different therapeutic approaches [1,2,3,4]. LUS also allows us to differentiate among different types of effusion, to quantify the effusion size, and to follow up on the response to medical therapy [2,3]. In addition, LUS does not expose patients to ionizing radiation, providing yet another advantage [1,2,3,4].

Despite these advantages, the role of LUS in children undergoing cardiac surgery remains surprisingly limited [2] compared to other pediatric settings [5,6,7,8,9,10], probably due to cultural inheritance mostly relying on chest radiography. The aim of this review is to provide a comprehensive overview and list of current potential applications for LUS in children with congenital heart disease (CHD), post-surgery, with the hope of encouraging its use for this important patient population. 

## 2. LUS Examination Protocols

LUS examinations are performed with either phased array probes or linear/convex probes. In neonates and children, linear and convex probes are preferred, however, as they offer a quick and comprehensive view of the entire lung field. 

**Figure 1:**
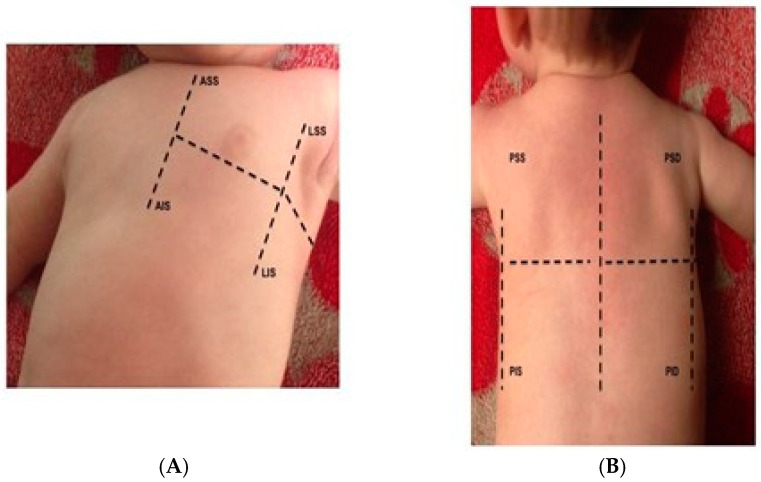
Six segments score. Each hemithorax is divided into 3 major quadrants (anterior, lateral, and posterior). Each quadrant is further subdivided into the upper and the lower half. (**A**) anterior and lateral, (**B**) posterior quadrants.

LUS examinations in adult patients should include the evaluation of different pulmonary areas and be performed in different views and positions. According to standardized protocols [11], for each hemithorax, 2 or 3 major areas (anterior, lateral, and sometimes posterior) delineated by the parasternal, anterior axillary, and posterior axillary line, respectively, should be identified and scanned. Each area can be further divided into an upper and lower half, creating 4 to 6 different quadrants for each hemithorax, namely anterior superior, anterior inferior, lateral superior, lateral inferior, posterior superior, and posterior inferior [11] (Figure 1). 

In children undergoing cardiac surgery for CHD, the posterior view is crucial for the diagnosis of atelectasis/effusion, the most common post-surgical, pulmonary complications. In a study of over 138 examinations at different post-operative times in 79 children (median age 9.3 months), the posterior areas were found to be more sensitive than the anterior and lateral areas in the diagnosis of effusion or atelectasis [12]. Lungs may be scanned posteriorly starting from the diaphragm to differentiate the lung from the liver, with a continuous brush up to the apex. The posterior view, however, may be not feasible to acquire in unstable children, particularly those with an open sternum, poorly cooperative children, or children with poor mobilization. In studies from our group, the posterior area was precluded in 7% of cases, while the anterior area could not be assessed in 11% due to bandages and medications covering a substantial part of the hemithorax. In contrast, the lateral area, despite the frequent presence of drainage tubes and other physical impediments, was almost always accessible (e.g., feasibility 98%) for LUS examination [12,13].

## 3. When and Who Should Perform LUS

There has been great debate on which members of the care team should perform LUS. Theoretically, LUS should be performed by any physician who oversees the patient (e.g., anesthetist, cardiologist, surgeon). Some recent works [14,15] suggest that LUS can be performed by mid-level healthcare professionals, including nurses [15] and physiotherapists [14] for whom LUS represents a unique tool to guide treatment and monitor results [14].

## 4. Common Findings

### 4.1. B-Lines

B-lines are the sonographic sign of partial deaeration of the lung parenchyma [11]. In CHD patients with left-to-right, or bidirectional shunt, LUS has a high sensitivity (94%), specificity (96%), and diagnostic accuracy (95%) for the assessment of lung congestion from pulmonary overflow, compared to CT [16]. Furthermore, neonates with CHD more B-lines compared to their healthy counterparts [17]. The presence of B-lines is almost universal after pediatric cardiac surgery due to extravascular fluid accumulation (particularly after cardiopulmonary bypass) and other effects of the main cardiac defect and post-surgical imbalance on the lung [4,12,13].

### 4.2. Classification of Lung Congestion in Children

**Figure 2:**
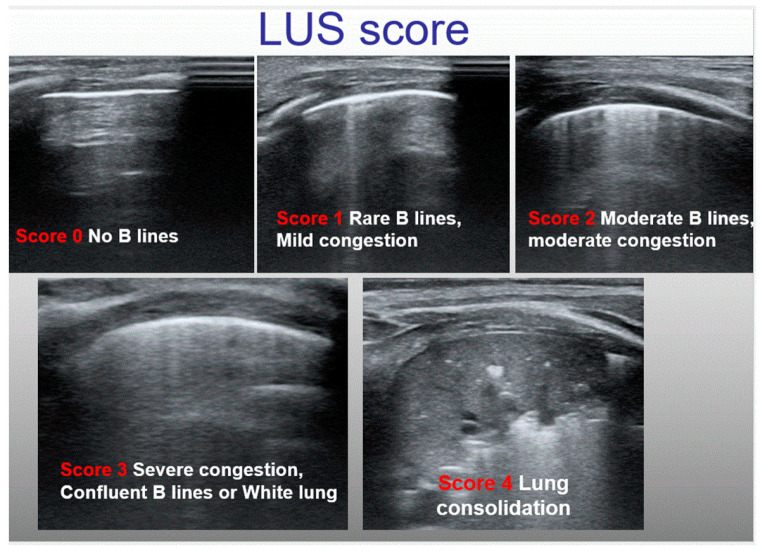
An example of semiquantitative LUS score that we have recently validated.

In each scanning area, B-lines are counted, and a score can be assigned for either single quadrants or for the entire hemithorax, the latter quantified by summing partial scores for each single scanning area. In adults, the main scores for the classification of lung congestion in heart failure are either the sum of B-lines in each area or the number of areas with more than three B-lines [18]; lung involvement is commonly classified into four categories (none, mild, moderate, and severe) [18,19]. In children, however, we use simplified scores; either qualitative or semiquantitative scores have been adopted. Some authors have proposed semiquantitative scores [13,17] (Table 1), while others [1,20] have proposed a qualitative score identifying three different patterns: (A) white lung, defined as the presence of confluent B-lines in two or more of the four areas; (B) the prevalence of B-lines in two or more areas; and (C) the prevalence of A-lines, or no significant congestion/normal lung (Figure 2). 

### 4.3. Pleural Effusion

**Figure 3:**
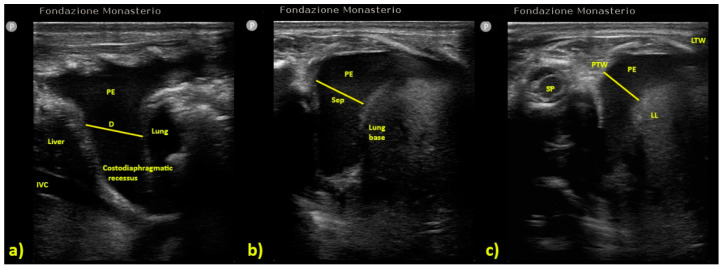
Formulas for pleural effusion quantification according to different authors: (**a**) Usta et al. [24], PEV is calculated by the formula D (mm) × 16, (**b**) Balik et al. [23] PEV is calculated by the formula Sep (mm) × 20; (**c**) Eibenberger [25] the major effusion’s diameter (D) is associated with PEV on a progressive scale (e.g., 10 mm correspond to 50–300 mL of PEV, 20 mm to 150–310 mL, etc.). (Table 2) D = distance; IVC = Inferior Vena Cava; LL = Lung Lower Lobe; LTW = Lateral Thoracic Wall; PE = Pleural Effusion; PEV = Pleural Effusion Volume; PTW = Posterior Thoracic Wall; Sep = maximal distance between parietal and visceral pleura; SP = Spine.

The evaluation of pleural effusion is one of the most employed applications of LUS. LUS has high sensitivity and specificity (93%) for the diagnosis of pleural effusion, comparable to computed tomography (CT), which remains the diagnostic gold standard but is invasive, time consuming, expensive, and exposes the children to dangerous radiations [2,3]. In addition, LUS may allow us to differentially diagnose the nature of post-surgical pleural effusion, highlighting yet another advantage. For instance, while anechoic effusion may be either a transudate or an exudate, the presence of internal echoes is highly suggestive of an exudate or a hemothorax [21,22,23,24,25,26,27,28]. Despite these advantages, the application of LUS for pleural effusion is often qualitative, classified as mild, moderate, or severe. Furthermore, there is lack of consensus for the quantitative measurement of pleural effusion by LUS. In adults, various algorithms, each using different projections and measurement methods, have been proposed for pleural effusion quantification [21,22,23,24,25,26,27,28], though none of these have been validated for infants and children (Table 2 and Figure 3). 

### 4.4. Atelectasis, Pneumonia, Consolidations, and Others

One of the main advantages of LUS over chest X-ray (CXR) is the ability to differentiate between effusion and atelectasis, which, as emphasized, are the most common pulmonary complications after pediatric cardiac surgery. Atelectasis is a universal complication after general anesthesia, either with tracheal intubation or laryngeal mask, occurring in about 68–100% of all types of surgery [29,30,31,32,33,34], with varying degrees of severity, ranging from small atelectasis to complete lung collapse. In these cases, the etiology of atelectasis is multifactorial and includes surgical compression, cardiopulmonary bypass, consequences on the lung of cardiac defect, and inappropriate ventilation. In children without cardiac defects or with no history of pulmonary disease undergoing minor surgery [29,30,31,32,33,34], atelectasis tends to resolve spontaneously, though it may persist after 3 days in about 70% of patients undergoing pediatric cardiac surgery [29]. 

LUS allows for the precise identification of regions of atelectasis. In several studies employing LUS after pediatric cardiac surgery, atelectasis was found to occur much more frequently in the inferior-posterior region (60–92.7%) than in the anterior (5–20.7%) or lateral regions (5–13.8%) [12,34]. LUS further helps in the differentiation of different types of consolidations and masses. Consolidations may be due to infection, infraction from pulmonary embolism, primary or metastatic cancer, compressive or obstructive atelectasis, or a contusion from thoracic trauma, all of which can potentially be differentiated by LUS [12,34]. For instance, the use of LUS for the diagnosis of pneumonia is today accepted in many NICUs, with diagnostic criteria based on major signs, including consolidation, air bronchograms, and pleural effusion. A recent (2020) meta-analysis [35] of over 22 studies with a total of 2470 patients demonstrated that LUS has high sensitivity (0.95; 95% CI: 0.94 to 0.96), specificity (0.90; 95% CI: 0.87 to 0.92), and diagnostic odds ratio (137.49; 95% CI: 60.21 to 313.98) for the diagnosis of pneumonia in children. 

### 4.5. Pneumothorax

**Figure 4:**
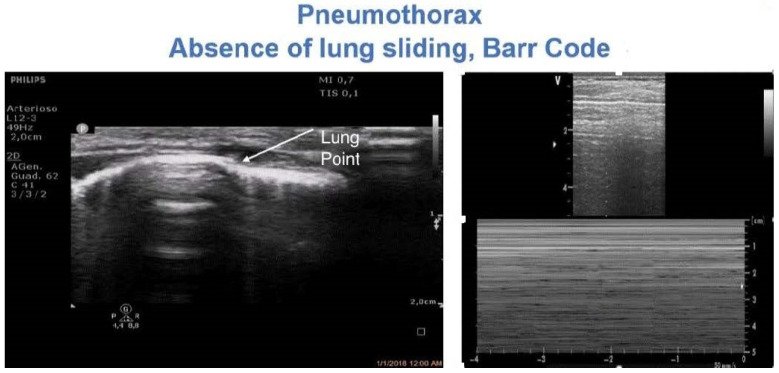
And Video S1: Diagnosis of pneumothorax. The lung pointy (e.g., the point where the pleura stops its movement) is highlighted on the left side. On the right side, the typical Barcode sign on M-mode is shown.

Pneumothorax is a common complication in cardiac surgery. Pneumothorax is diagnosed by LUS based on three major findings, namely the absence of lung sliding and lung pulse, the absence of B-lines, and evidence of the ‘lung point’ [1,36]. The first two signs are required, whereas the lung point may not always be detectable. LUS revealed optimal diagnostic accuracy, with superior sensitivity and similar specificity compared to CXR for the detection of pneumothorax, and it was found to be superior to CT in the classification of pneumothorax size [1,36]. Thus, LUS may be extremely useful for the diagnosis of pneumothorax in a pediatric cardiac surgery setting [13,37]. LUS may be used to monitor the occurrence of pneumothorax after drainage removal, avoiding serial CXRs as is routine practice in many centers [37] (Figure 4 and Video S1). 

**Table 2:** Major studies proposing formula for pleural effusion quantification.

Authors	Population	Protocol of Examination	Formula
Remérand et al. [22], France	58 (45 M) Age 58 ± 17 years	SUPINE Transverse views positioning the transducer in each IS. The transducer was slipped between the patient’s back and mattress. The lower and upper IS where PE was detected were drawn on the patient’s skin PE length measured in paravertebral regions between the apical and caudal limits. Cross-sectional area measured at the mid-length of PE	PEV (mL) = ACT (cm^2^) × LCT (mm)
Usta [24], Germany	135 (90 M) Age 60 (45–67) years	SITTING The transducer was moved in a cranial direction in the mid-scapular line. PE diameter: maximal distance between mid-height of the diaphragm and visceral pleura	PEV (mL) = D (mm) × 16
Balik et al. [23], Czech Republic	81 (47 M) m. ventilated patients Age 60 ± 15 years	SUPINE The transducer was moved in the cranial direction in the posterior axillary line PE diameter: maximal distance between parietal and visceral pleura at the lung base	PEV (mL) = 20 × Sep (mm)
Eibenberger [37], Austria	51 (21 M) Age 28–82 years	SITTING Latero-dorsal wall of the chest PE diameter: the maximal perpendicular distance between the posterior wall of the lung and the posterior chest wall	D (mm) PEV (mL) 0 0–90 10 50–300 20 150–310 30 160–660 40 490–1670 50 650–1840 >60 950–251
Vignon et al. [21], France	97 (61 M) age 59 ± 20 years	SUPINE From the base to the apex of the chest, along the dorso-lateral part of the chest wall, as far as possible posterior between the mattress and the patient’s back without lifting the hemithorax. PE diameter: the maximal distance from the leading edge of the dependent surface of the lung to the trailing edge of the posterior chest wall, on transverse views of pleural spaces. Measurements were made at the base and at the apex of the pleural space	D > 45 mm at the RTB D > 50 mm at the LTB base predicted a PEV ≥ 800 mL sensitivity of 94% and 100 and specificity of 76 and 67%, respectively

ACT: pleural effusion cross-sectional area; EE: end-expiration; EI: end-inspiration; IS: inter-costal space; LCT: pleural effusion length; LTB: left thoracic base; m.: mechanical; PEV: pleural effusion volume; RTB: right thoracic base; Sep: separation; V: volume; D: diameter; PE: pleural effusion; BMI: body mass index. A Typically, the inter-pleural distance was greater at end-expiration in ventilated patients and on inspiration in spontaneously breathing patients.

### 4.6. The Retrosternal Area: Diagnosis of Clots

To conclude our discussion of the LUS examination, we found it useful to explore the inspection of the parasternal region for the evaluation of the retrosternal area, a zone where clots are commonly known to form after pediatric cardiac surgery [2,28,38].

By placing the probe close to the parasternal line, the anterior segments can be scanned up and down. If a clot or hematoma is detected, the probe should be placed over it and freely tilted in various planes or orientations for visualization. 

There is no standardized system to measure and classify clot dimensions. In a recent series, we defined clot size according to the maximal diameter on an axis perpendicular to the cardiac wall as follows. We specifically defined four classes of clot size, namely (1) large clots: >3 cm; (2) moderate sized clots: >2 to <3 cm; (3) small to moderate sized clots >1 to <2 cm; and (4) small clots: <1 cm.

Among 37 children undergoing total cavopulmonary connection (mean age 5.5 ± 1.8 years, (range 2.4–11.7) (2.38) mean body surface area 0.7 ± 0.1 m^2^ (range 0.3–1.6 m^2^)), retrosternal clots were detected in 18 children (48.6%). Of these, seven (13.5%) had small clots (<1 cm), two (5.4%) had small to moderately sized clots (>1–<2 cm), three (8.1%) had moderately sized clots (>2–<3 cm), and six (16.2%) had large clots (>3 cm). Four of the six detected large clots required surgical revision, and the other two clots were not treated because the patients were clinically stable.

**Figure 5:**
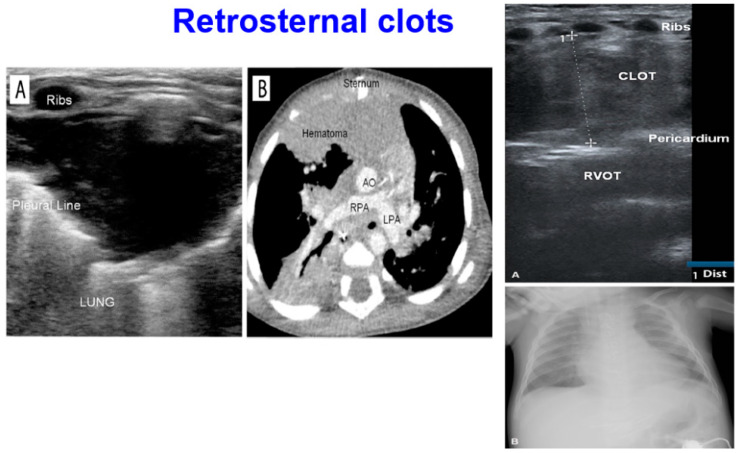
Retrosternal clot. (**Left**) In (**A**), a retrosternal clot is visualized by LUS and confirmed (**B**) by CT scan. Using LUS, it is possible to appreciate how the clot is interposed among the sternum and the plural line. (**Right**) A retrosternal clot among the strum and the right ventricular outflow tract (RVOT) is visualized by LUS (**A**). On chest X-ray and enlargement of right mediastinum can be observed (**B**).

Furthermore, exploring the retrosternal area may be helpful for the diagnosis of serious complications after cardiac surgery such as mediastinitis [28], which is typically characterized by retrosternal fluid collection and parasternal hyperconvexity. Hematoma and infections may have similar finding and may overlap [28]; thus, echographic findings should always be correlated clinically (Figure 5).

### 4.7. Diaphragmatic Motion Anomalies

**Figure 6:**
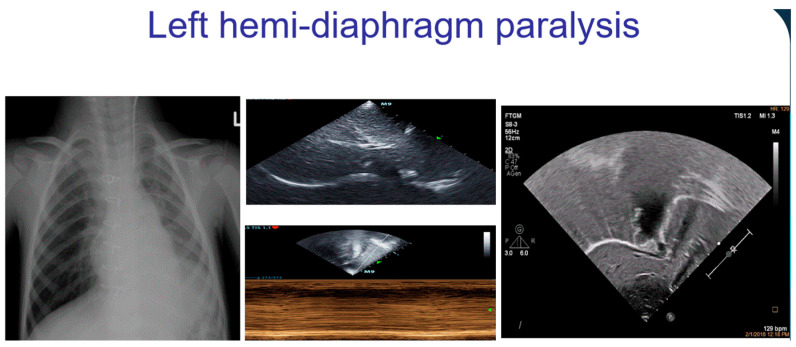
And Video S2: A left hemidiaphragm paralysis can be visualized by chest X-ray (left side) and confirmed by echographic analysis of diaphragm by subcostal view. In the middle, a paradoxical motion of the diaphragm can be appreciated in M-mode, and on the right image, the left hemidiaphragm lifted can be appreciated (Video S2).

Diaphragmatic paralysis is a serious complication after pediatric cardiac surgery and occurs in 0.3–12.8% of patients. Consequences of diaphragmatic paralysis include respiratory insufficiency, pulmonary infections, and the prolongation of hospital stay. Diaphragmatic paralysis is usually associated with concomitant atelectasis [12,39,40] and may be easily diagnosed either with LUS or with conventional echocardiography by subcostal view. Diagnosis is confirmed by comparing each hemidiaphragm in subxiphoid view and evaluating their respective movements using M-mode. Diaphragmatic motion can be classified as normal (towards the transducer in inspiration with a difference of excursion between the hemidiaphragms of <50%), decreased (difference in the amplitude between the hemidiaphragms >50%), absent (flat line at M-mode), or paradoxical (with absent and paradoxical motion away from the transducer in inspiration), the latter indicating diaphragmatic paralysis [12,39] (Figure 6 and Video S2). 

### 4.8. LUS Guidance of Interventional Procedures

LUS may be used to guide common interventional procedures in pediatric cardiology, including drainage insertion for pleural effusion and pneumothorax [41]. Adult studies have demonstrated that the routine use of LUS may drastically reduce the risk of pneumothorax in thoracentesis from 8.8% to 0.97% (*p* < 0.0001) [41]. The utility of LUS extends to tracheal tube verification in the NICU [42,43]. The echographic visualization of the tracheal tube tip by LUS was found to be feasible (i.e., 83% to 100%) and had good sensitivity (i.e., 0.91 to 1.00) with sufficient specificity (i.e., 5 to 1.0) for appropriate tracheal tube depth verification. Furthermore, LUS may be used for echo-guided lung recruitment [3].

### 4.9. Chest X-ray Reduction in Pediatric Cardiac Surgery

In a previous study, we analyzed [44] the medical records of 1487 children and adolescents (7.09 ± 12.34 years, range 0–17 years) who underwent cardiac surgery over a 6-year period (2013–2018) at our Center, to assess whether the systematic use of LUS reduces the use of CXR. We retrospectively compared CXR use between 2013–2015, where LUS was not routinely employed, with CXR utilization between 2016–2018, after the introduction of systematic LUS use. We found a significant reduction in the number of chest radiographs (10.68 ± 10.31, *p* < 0.005), corresponding to a radiation dose reduction of 0.032 mSv for each individual patient. 

### 4.10. Prognostic Utility of LUS

More recently, several studies evaluated not only the diagnostic capabilities but also the prognostic potential of LUS in pediatric cardiac surgery [1,13,45]. Vitale et al. showed that 20 children (<20 kg; 3–7.25 months) with higher pulmonary congestion on day one post-op had longer times on cardiopulmonary bypass (CPB), longer cross clamp times, longer need of mechanical ventilation, and lengthened stay in ICU [1]. In another study of 61 children (3 days–7.4 years), the percentage of B-lines 1–6 h postoperatively predicted the length of mechanical ventilation and PICU stay [45]. The incremental prognostic value of a new LUS score post-cardiac surgery has been demonstrated. In one study of 237 children undergoing cardiac surgery (0–17 years) at a single center, the use of a new LUS score 12–36 h post-surgery better predicted the intensive care length of stay (beta 0.145; *p* = 0.047) and extubation time (beta 1.644; *p* = 0.024), compared with conventional risk factors. Of note, when single quadrants were analyzed, only the anterior LUS score had significant prognostic value (ICU stay beta, 0.471; *p* = 0.020; extubation time beta 5.530; *p* = 0.007).

## 5. Current Gaps of Knowledge 

### 5.1. Why Is the Lung White?

**Figure 7:**
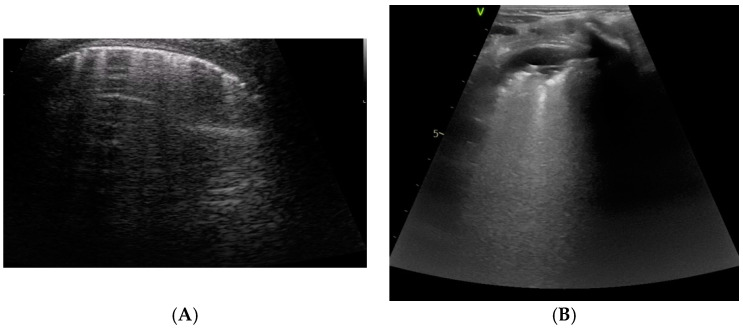
(**A**) B-lines due to cardiogenic lung congestion (regular, uniformly distributed along the lungs, with a regular pleural line) and (**B**) B-lines typical of a lung disease (irregular, patchy, with altered pleural line) (Video S3).

In several cases, deaeration, or white lung, can occur due to extravascular water content in response to hypoxia. Many children with CHD, both before and after surgery, present cases of pulmonary edema, due to increased pulmonary blood flow, ventricular dysfunction, valve defects, etc., and deaeration from chronic hypoxia or recovery from atelectasis [2,3]. Pulmonary atelectasis commonly occurs after pediatric surgery and/or in the ICU, and if scanned during post-operative recovery, it is difficult to differentiate from severe pulmonary congestion. Characteristic B-lines help to differentiate cardiogenic lung congestion from other forms of lung deaeration. In cardiogenic lung congestion, B-lines are uniformly present on either hemithorax with a gravity-dependent distribution, with thin and regular pleural lines [11,18] (Figure 7A and Video S3). A patchy, irregular distribution of B-lines, often with irregular pleural lines, is more characteristic of non-cardiogenic pulmonary edemas, such as is observed in acute respiratory distress syndrome (ARDS) or pulmonary fibrosis [11,18] (Figure 7B).

### 5.2. Future Perspectives

Studies on adult patients suggest that the use of speckle tracking may improve the accuracy for the diagnosis of pneumothorax [46,47]; however, as mentioned above, the data on such applications in pediatric populations are lacking.

Preliminary observations in children suggest that the use of contrast agents is feasible and may increase the accuracy for the diagnosis of complicated pneumonia, accurately differentiating necrotizing pneumonia from complex parapneumonic effusion [48]. The use of contrast agents may further allow us to accurately visualize the drainage tubes during invasive maneuvers such as drain insertion.

Studies of diaphragmatic structure and motion, including the quantification of diaphragm thickness, diaphragm excursion, and diaphragm thickening, are increasing in relevance for both the diagnosis of post-surgical paralysis and the monitoring of pulmonary recovery and response to therapy [49,50].

## 6. Conclusive Remarks

LUS is an accurate, fast, cheap, and radiation-free tool that may be employed for the diagnosis and follow-up of major pulmonary complications in pediatric cardiac surgery. The systematic use of LUS in pediatric cardiology should be encouraged to reduce serial CXR examinations that are not only expensive but also expose children to potentially high doses of radiation. 

Further studies are warranted to establish a consensus classification system for the evaluation of disease severity and to further assess the prognostic potential of LUS in children undergoing cardiac surgery for CHD.

## Figures and Tables

**Table 1:** Major semiquantitative scores for lung congestion classification in children.

Authors	Classifications
Wu (34)	(I) Normal: A lines (II) Mild: fewer than 3 B lines in 2 rip spaces with spared areas (III) Moderate: between 3 and 7 B lines between 2 rip spaces (IV) Severe: more than 7 or coalescent B-lines from the base to the apex without spared area
Cantinotti (12,13)	(I) trivial-none (LUS-score = 0–6), (II) mild (LUS-Score = 6–12), (III) moderate (LUS-Score = 13–24) (IV) severe (LUS-Score > 24)
Raimondi, Vitale (15,20)	(I) Type 1- full hyperechoic image of the lung fields or ‘white lung’; (II) Type 2- prevalence of B lines, that is, vertical, comet-tail artifacts. (III) Type 3- predominance of A lines

## Supplementary Materials

The following supporting information can be downloaded at: https://www.mdpi.com/article/10.3390/diagnostics12030763/s1, Video S1: pneumothorax, Video S2: diaphragmatic paralysis, Video S3: A lines cardiogenic.
